# Primary pleomorphic liposarcoma of the liver: a case report and literature review

**DOI:** 10.1186/s40792-021-01322-4

**Published:** 2021-11-19

**Authors:** Yuri Terunuma, Kazuhiro Takahashi, Manami Doi, Osamu Shimomura, Yoshihiro Miyazaki, Kinji Furuya, Shoko Moue, Yohei Owada, Koichi Ogawa, Yusuke Ohara, Yoshimasa Akashi, Shinji Hashimoto, Tsuyoshi Enomoto, Tatsuya Oda

**Affiliations:** 1 College of Medicine, School of Medicine and Health Sciences, University of Tsukuba, Tsukuba, Japan; 2 Department of Gastrointestinal and Hepatobiliary Pancreatic Surgery, University of Tsukuba, Tennoudai 1-1-1, Tsukuba, 3059575 Japan

**Keywords:** Liposarcoma, Primary hepatic liposarcoma, Pleomorphic liposarcoma, Liver, Case report

## Abstract

**Background:**

Primary liposarcoma arising from the liver is exceedingly rare. There have been very few reports documenting primary hepatic liposarcoma, especially of the pleomorphic subtype. Surgery is currently the only established treatment method, and the prognosis remains poor. In this report, we present an unusual case of hepatic liposarcoma of the pleomorphic subtype with literature review. In addition, we discuss theories regarding pathogenesis and the pathological and clinical features of primary hepatic liposarcoma to better outline this rare entity.

**Case presentation:**

An asymptomatic 65-year-old female was found to have a right hepatic mass on a computed tomography scan 2 years after surgical resection of the left adrenal gland and kidney for adrenocortical carcinoma. Laboratory examinations were unremarkable. Magnetic resonance imaging demonstrated a 16-mm mass in the right hepatic lobe. Adrenocortical carcinoma metastasis was suspected. Laparoscopic partial hepatectomy completely removed the tumor with clear margins. Macroscopically, the surgical specimen contained a nodular, yellow–white mass lesion 20 mm in diameter. On pathologic examination, pleomorphic, spindle-shaped tumor cells containing hypochromatic, irregularly shaped nuclei of various sizes formed fascicular structures. Scattered lipoblasts intervened in varying stages. Mitotic cells were frequent. Ki-67 labeling index was 15%. Immunohistochemically, the tumor cells were diffusely positive for vimentin and focally positive for CD34 and alpha-SMA; lipoblasts were focally positive for S-100. Tumor cells were nonreactive for SF-1, inhibin alpha, desmin, HHF35, HMB45, Melan A, MITF, c-kit, DOG1, cytokeratin AE1/AE3, h-caldesmon, STAT6, CD68, MDM2, CDK4, c17, DHEAST, 3BHSD, CD31, Factor 8, and ERG. From these findings, primary hepatic liposarcoma of pleomorphic subtype was diagnosed. The tumor recurred intrahepatically 3 years later, and the patient died 5 months after recurrence.

**Conclusions:**

In our report, we discussed the rarity, theories regarding pathogenesis, and a review of the literature of this atypical condition. To the best of our search, this is the 14th case of primary hepatic liposarcoma and the 2nd case of the pleomorphic subtype reported throughout the world. Further research regarding the etiology of this unusual clinical entity is warranted to establish effective diagnostic and management protocols.

## Background

Liposarcoma is a rare malignant adipocyte tumor derived from mesenchymal cells. Representing approximately 15–20% of all soft tissue tumors, liposarcoma usually develops in the extremities and retroperitoneum [1]. In contrast, primary occurrence in the liver is remarkably rare. Histologically, liposarcoma has five distinct subtypes: atypical lipomatous tumor/well-differentiated, dedifferentiated, myxoid, pleomorphic, and myxoid pleomorphic. Among the five subtypes, pleomorphic liposarcoma is exceptionally rare and accounts for < 5% of primary liposarcomas [2]. Currently, surgery is the only established method of treatment for liposarcoma. The prognosis remains extremely poor, especially for the pleomorphic subtype.

In this report, we present a rare case of primary pleomorphic liposarcoma originating from the liver with literature review. Due to the particular rarity of the pleomorphic variety, very few cases have been reported thus far. In addition, we discuss theories regarding pathogenesis and the pathological and clinical features of primary hepatic liposarcoma to better outline this rare entity.

## Case presentation

A 65-year-old Japanese woman was found to have a heterogeneous, ring-enhancing, 11-mm mass in the right hepatic lobe on follow-up contrast-enhanced abdominal computed tomography (CT) 2 years after surgical resection of the left adrenal gland and kidney for left-sided adrenocortical carcinoma. Medical and family histories were negative for hepatic disorders. The patient reported no symptoms and was subsequently referred to our department for further evaluation and treatment.

**Fig. 1:**
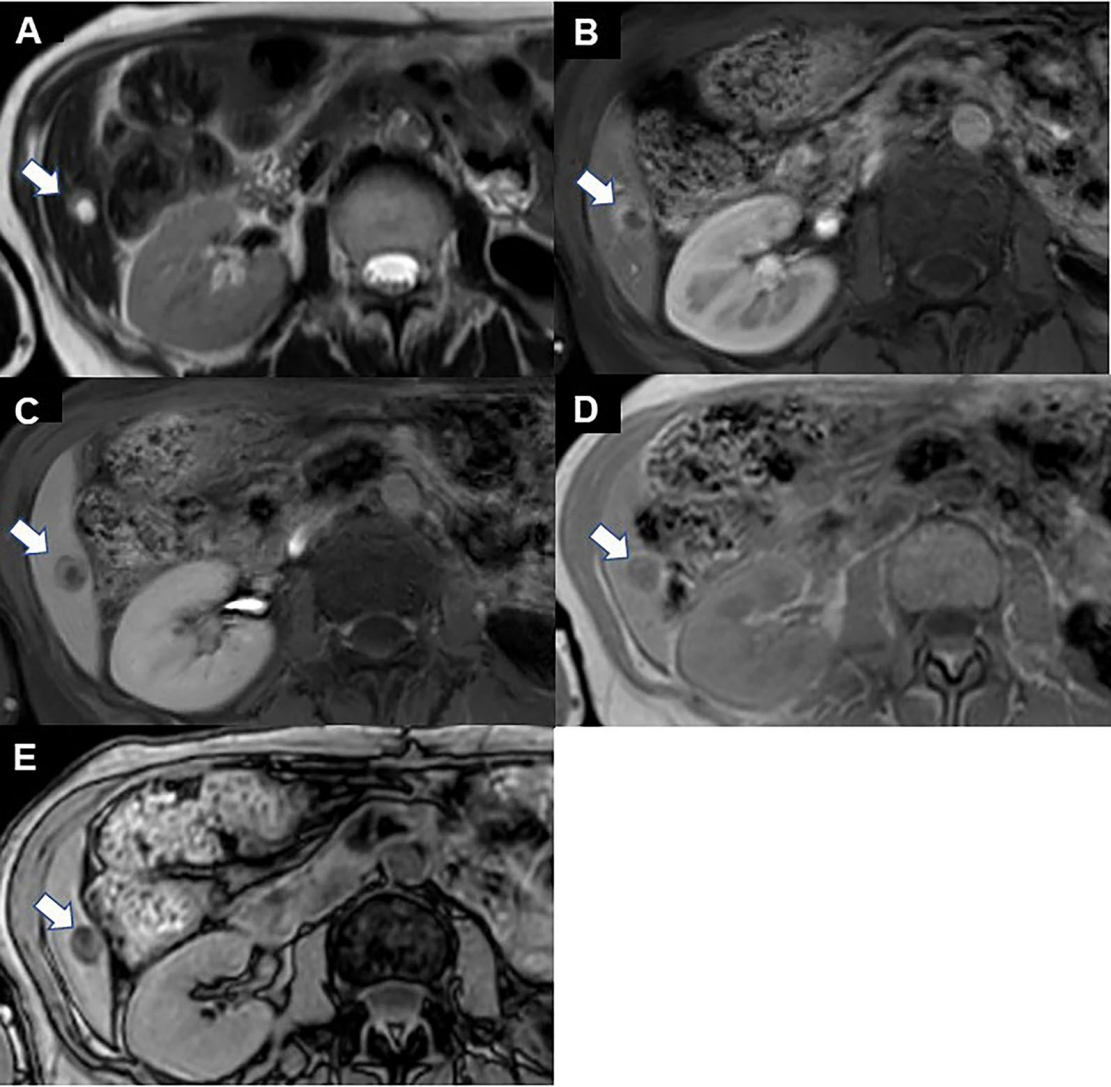
Dynamic contrast-enhanced MRI of the abdomen. **A** A 16-mm mass lesion with both cystic and solid, lipid-containing components (arrow, T2WI). **B** Arterial phase (T1W1). **C** Portal phase (T1W1). The solid components demonstrated arterial enhancement followed by rapid washout in the portal phase (arrow, T1WI). **D** In-phase (T1W1). **E** Opposed-phase (T1W1). A signal drop can be observed on the opposed-phase image compared to the in-phase image (arrow)

On admission, physical examination revealed a well-appearing woman in no acute distress. Laboratory examinations were unremarkable. Serologic testing for hepatitis B and C was negative. Alpha-fetoprotein and des-γ-carboxy prothrombin levels were within normal limits. Dynamic contrast-enhanced T2-weighted images (T2WI) on magnetic resonance imaging (MRI) of the abdomen showed a 16-mm mass lesion with both cystic and solid, lipid-containing components (Fig. 1A). The solid components demonstrated arterial enhancement followed by rapid washout on T1-weighted images (T1WI) (Fig. 1B, C). Although this description suggested hepatocellular carcinoma (HCC), a lack of cirrhotic changes and viral hepatitis made HCC unlikely. A signal drop was observed on opposed-phase T1WI compared to in-phase T1WI, indicating the presence of lipids (Fig. 1D, E). No other intraabdominal, peritoneal, or retroperitoneal masses were noted. Based on these findings and the patient’s history, adrenocortical carcinoma metastasis was suspected. Laparoscopic partial hepatectomy was scheduled. The operation was successfully executed as planned. The tumor was completely resected with clear surgical margins.

**Fig. 2:**
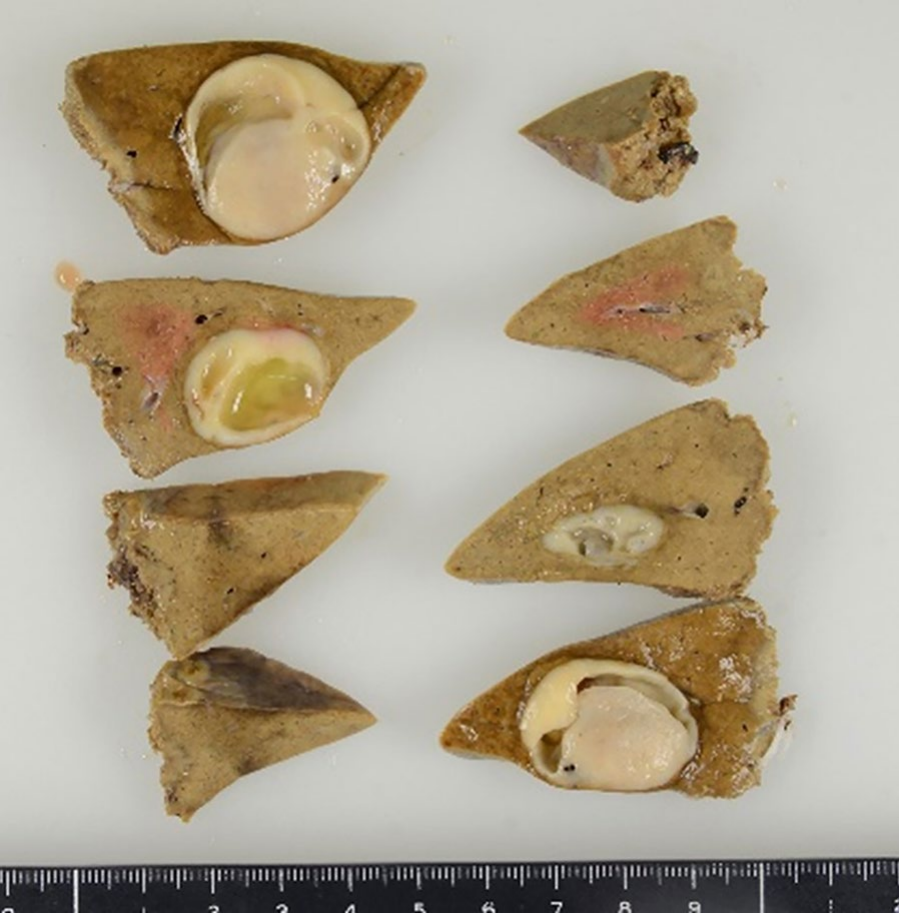
Macroscopic of the resected specimen. The surgical specimen contained a nodular, yellow–white mass lesion with clear borders 20 mm in size. The cut plane was smooth and glossy with a slight bulge

Macroscopically, the surgical specimen contained a nodular, yellow–white mass lesion with clear 20-mm borders. The cut plane was smooth and glossy with a slight bulge (Fig. 2).

**Fig. 3:**
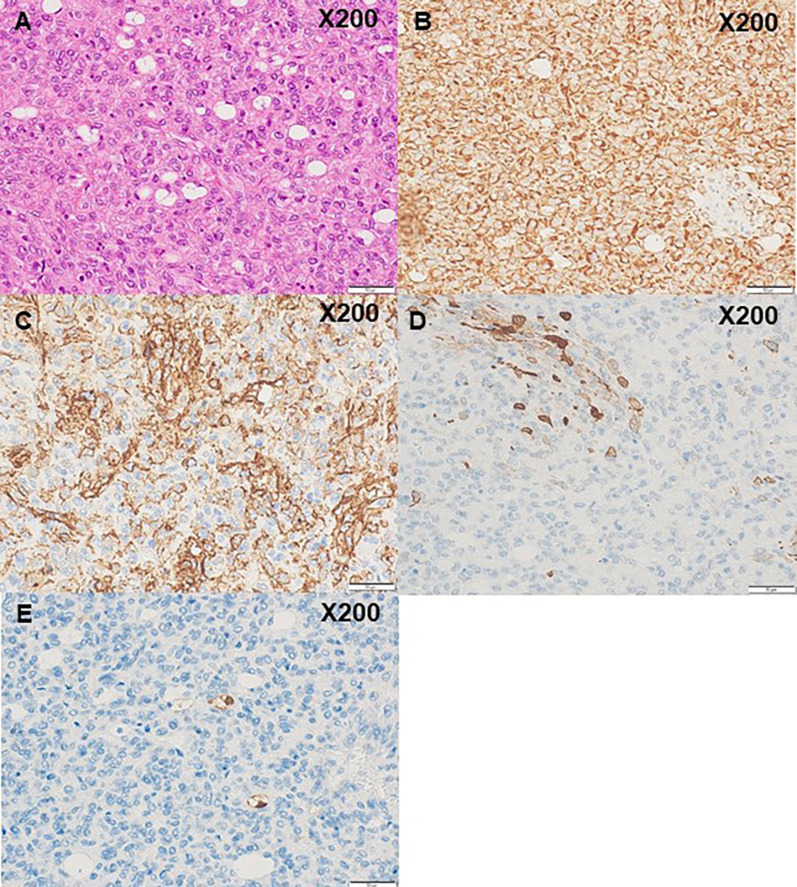
Microscopic findings of the tumor. **A** Hematoxylin & Eosin staining (× 200). The tumor was composed of pleomorphic, spindle-shaped cells forming fascicular structures with scattered lipoblasts intervening in varying stages. **B** Vimentin, **C** CD34 staining **D** alpha-SMA staining **E** S-100 staining. Immunohistochemically, tumor cells were diffusely positive for vimentin and focally positive for CD34 and alpha-SMA. Lipoblasts were focally positive for S-100

Microscopically, the tumor comprised pleomorphic, spindle-shaped cells forming fascicular structures with scattered lipoblasts intervening in varying stages (Fig. 3A). The tumor cells contained hypochromatic, irregularly shaped nuclei of various sizes. There was also capillary growth between cells in a pericytomatous pattern. Mitotic cells were frequent. Ki-67 labeling index was 15%. Immunohistochemically, the tumor cells were diffusely positive for vimentin and focally positive for CD34 and alpha-SMA (Fig. 3B–D); lipoblasts within the tumor were focally positive for S-100 (Fig. 3E). In contrast, the tumor cells were nonreactive for SF-1, inhibin alpha, desmin, HHF35, HMB45, Melan A, MITF, c-kit, DOG1, cytokeratin AE1/AE3, h-caldesmon, STAT6, CD68, MDM2, CDK4, c17, DHEAST, 3BHSD, CD31, Factor 8, and ERG. Fluorescent in situ hybridization (FISH) findings with a split signal probe for DDIT3, CREB1, and ATF1 showed split signals of 2%, 2%, and 0%, respectively. These collective findings support the diagnosis of pleomorphic liposarcoma as described by the World Health Organization (WHO) Classification of Tumours: Soft Tissue and Bone Tumours, 5th edition [2].

The postoperative course was uneventful. The patient did not receive adjuvant chemotherapy or radiotherapy and was followed with abdominal CT scans every 3–6 months. Abdominal CT taken 41 months after surgery revealed a 104-mm heterogeneous mass spanning the anterior, medial, and posterior liver segments. Repeat imaging 1 month later demonstrated rapid growth to 141 mm in diameter. Needle biopsy indicated liposarcoma pathologically, which was compatible with the original tumor. The patient received right hepatectomy. Histopathological examination confirmed pleomorphic liposarcoma recurrence. Despite these extensive methods, however, new liver recurrences, lung metastases, and peritoneal dissemination of multiple small nodules were noted on a CT scan 1 month later. Palliative chemotherapy with eribulin was initiated. A CT scan taken 1 month into chemotherapy showed rapid enlargement of the lesions consistent with progressive disease. Best supportive care was started and continued until the patient died 3 months later.

## Discussion

**Fig. 4:**
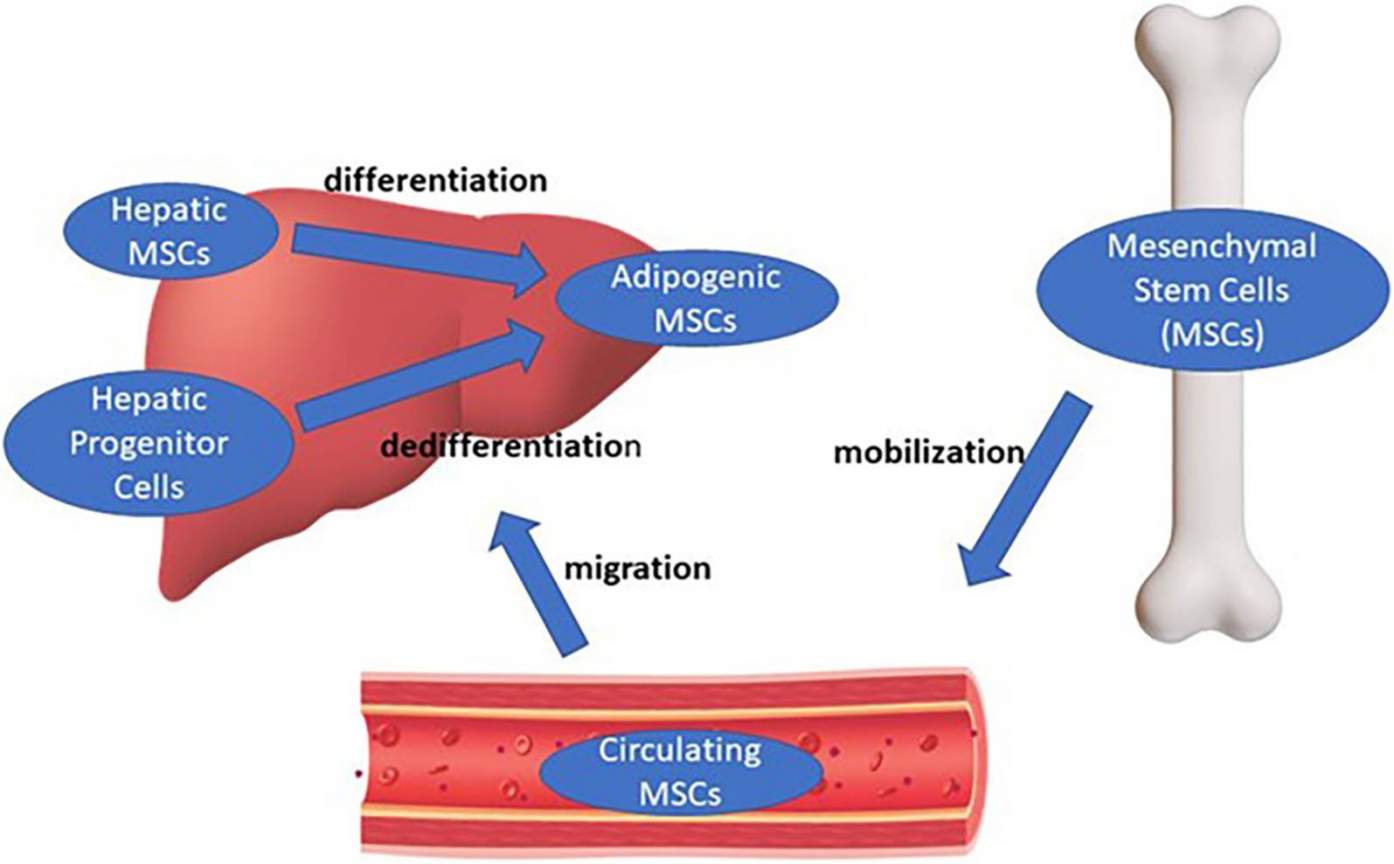
Potential source of adipocytic cells in primary hepatic liposarcoma. Hepatic mesenchymal stem cells (MSCs), circulating MSCs, and hepatic progenitor cells (HPCs) are potential origins. Hepatic MSCs serve as potential cells of origin for primary hepatic liposarcoma due to their multipotency. Stimulation or damage to the liver can trigger circulating MSC migration, and abnormal adipocytic differentiation may form hepatic liposarcoma. HPCs result from hepatocytes that dedifferentiate and undergo epithelial–mesenchymal transition to mesenchymal-like stem cells, which, in turn, form the basis of liposarcoma development

Primary liposarcoma is a malignant tumor of adipocytic differentiation. However, because the liver generally lacks adipocytes, the source of adipocytic cells in primary hepatic liposarcoma is unclear. Hepatic mesenchymal stem cells (MSCs), circulating MSCs, and hepatic progenitor cells (HPCs) are potential alternative origins (Fig. 4). Hepatic MSCs are multipotent cells that comprise only 0.001% of liver cells [3]. In addition to their ability to differentiate into hepatocytes, hepatic MSCs are capable of committing to various mesodermal lineages, including the adipogenic, osteogenic, myogenic, and chondrogenic lineages. Despite comprising a minuscule fraction of liver cells, hepatic MSCs serve as potential cells of origin for primary hepatic liposarcoma due to their multipotency. Circulating stem cells deploy into the bloodstream in a clinical process termed “mobilization” as a normal response to inflammatory cytokines [3]. These mobilized cells then migrate to damaged tissue, including the liver, and aid repair. Theoretically, liver damage can trigger circulating MSC migration, and abnormal adipocytic differentiation may form hepatic liposarcoma. HPCs arise from hepatocytes that dedifferentiate and undergo epithelial–mesenchymal transition [3]. Disruptions in this transition process can produce aberrant HPCs. These cells can, in turn, form the basis of liposarcoma development. In this patient, tumor cells were reactive for vimentin and alpha-SMA. These markers indicate MSC origin [3]. In addition, the tumor cells were also positive for CD34, a marker present on both hematopoietic stem cells and HPCs [3]. The findings from our patient support hepatic MSCs, circulating MSCs, or HPCs as possible cells of origin for primary hepatic liposarcoma, although definitive etiology cannot be determined from these results alone.

In 2020, the WHO renewed its classification criteria for liposarcoma to recognize five distinct subtypes based on pathological features [1, 2]. The pleomorphic subtype is particularly uncommon, accounting for < 5% of all cases of liposarcoma [2]. Histologically, pleomorphic liposarcoma resembles high-grade undifferentiated sarcoma containing variable numbers of pleomorphic lipoblasts [2]. Immunohistochemically, tumor cells may be focally positive for alpha-SMA, while pleomorphic lipoblasts show variable positivity for S-100 [4, 5]. Tumor cell reactivity for cytokeratin, epithelial membrane antigen, and desmin also varies [4, 5]. In the present case, pathological inspection revealed lipoblasts and pleomorphic, spindle-shaped tumor cells, raising the possibility of angiomatoid fibrous histiocytoma. The tumor was relatively rich in mature adipocytes, suggesting perivascular epithelioid cell tumor (PEComa) and well-differentiated liposarcoma as differential diagnoses. Because lipoblasts are the *sine qua non* of liposarcoma diagnosis, dedifferentiated liposarcoma, myxoid liposarcoma, and myxoid pleomorphic liposarcoma were also differential diagnoses. Other differentials included epithelioid gastrointestinal stromal tumor (GIST), solitary fibrous tumor (SFT), and angiosarcoma due to positive staining for CD34. Adrenocortical carcinoma metastasis was also considered from the patient’s past medical history. Immunohistochemically, the lipoblasts stained positive for S-100. The tumor cells were negative for desmin, HHF35, HMB45, Melan A, MITF, c-kit, DOG1, cytokeratin AE1/AE3, h-caldesmon, STAT6, CD68, CD31, Factor 8, and ERG, making diagnoses of PEComa, GIST, SFT, and angiosarcoma unlikely. The presence of lipoblasts further ruled out angiosarcoma. Negative staining of CDK4 and MDM2 ruled out well-differentiated liposarcoma and dedifferentiated liposarcoma. Furthermore, negative results for SF-1, inhibin alpha, c17, DHEAST, and 3BHSD ruled out adrenocortical carcinoma metastasis. Split-signal FISH results negative for significant DDIT3 signal splitting ruled against myxoid liposarcoma. Similarly, low split signals for CREB1 and ATF1 lowered the possibility of angiomatoid fibrous histiocytoma. Myxoid pleomorphic liposarcoma was ruled out from the lack of characteristic myxoid matrix and oval cells. From these pathological and immunohistochemical findings, the patient’s tumor was classified as a hepatic liposarcoma of pleomorphic subtype.

Pleomorphic liposarcoma is an aggressive neoplasm with an overall poor prognosis. According to previous studies focusing on pleomorphic liposarcoma from the orthopedic field [4, 5], the 5-year overall, metastasis-free, and local recurrence-free survival rates ranged from 57–63%, 50–58%, and 48–58%, respectively. Advanced age (> 60 years), large tumor size (> 10 cm), and truncal location are common indicators of poor clinical outcomes. Although radical surgical resection with clear margins is currently the treatment of choice for liposarcoma [6], there is no standardized treatment protocol. Adjuvant chemotherapy and radiotherapy are controversial for the pleomorphic subtype due to variable sensitivity [1]. Several molecular targeted therapies exist for other subtypes, including CDK4 inhibitors and MDM2 inhibitors for well-differentiated liposarcoma and dedifferentiated liposarcoma, and trabectedin for myxoid liposarcoma [1]. However, such disease-specific therapies are ineffective for pleomorphic liposarcoma due to its genetic heterogeneity compared to other subtypes [1]. Clinical trials regarding immunotherapy with anti-PDL1 or anti-CTLA4 antibodies are ongoing [7]. Our patient underwent right lobe hepatectomy for pleomorphic liposarcoma without ancillary chemotherapy or radiotherapy. Despite confirmation of negative surgical margins, the tumor recurred in the liver after 41 months. Even after repeating surgical excision, further liver recurrences and distant metastases to the lungs and peritoneum appeared 1 month later. These clinical results are in line with the aggressive nature of pleomorphic liposarcoma documented in previous reports.

A PubMed literature search yielded 13 previous cases of primary hepatic liposarcoma (Table 1) [6, 8–19]. Most of the patients were adults, although three were children. Tumor origins included the hepatic lobes and hilum. Pathologically, one case was atypical lipomatous tumor/well-differentiated, seven were myxoid, one was dedifferentiated, and one was pleomorphic. Surgical resection was the mainstay of treatment, although some patients received adjuvant chemotherapy or chemoradiotherapy with the Intergroup Rhabdomyosarcoma Study Protocol [12] (vincristine, actinomycin D, cyclophosphamide, and radiotherapy) or the MAID regimen [18] (mesna, adriamycin, ifosfamide, and dacarbazine). Of the nine cases that reported patient outcomes, tumor recurrence occurred in more than half of the patients in a short period, with a high mortality rate. Our case is the 14th reported case of primary hepatic liposarcoma in the world and the 2nd case of the pleomorphic subtype.

**Table 1:** Case reports of primary liposarcoma originating in the liver

Case	Author	Age/Sex	Location	WHO subtype	Treatment	Prognosis
1	Wolloch et al., 1973 [8]	22 y/F	Right lobe	Myxoid	Right lobe hepatectomy	Death 46 days after surgery
2	Kim et al., 1987 [9]	30 y/F	Left lobe	Dedifferentiated	Left lobe hepatectomy	NED 10 months
3	Chen et al., 1988 [10]	n/a	n/a	n/a	n/a	n/a
4	Soares et al., 1989 [11]	2 y/M	Hilum	n/a	Autopsy	n/a
5	Wright et al., 1993 [12]	3 y/M	Hilum	Myxoid	Surgical resection, chemotherapy, and radiotherapy	Local recurrence 11 years later; death 3 months after recurrence
6	Aribal and Berberoglu, 1993 [13]	48 y/F	Hilum	Myxoid	Chemotherapy	n/a
7	Khan et al., 2001 [14]	50 y/M	Right lobe	n/a	Right lobe hepatectomy	n/a
8	Nelson et al., 2001 [15]	54 y/F	Right and left lobes	Myxoid	Exploratory laparotomy with biopsy	Postoperative hemorrhage and death
9	Kuo et al., 2006 [16]	61 y/F	Right and left lobes	Myxoid	Three-segmentectomy (1st time); hepatectomy (2nd time); hepatectomy (3^rd^ time); radiotherapy	Repeated recurrences; death 26 months after initial diagnosis due to liver failure and aspiration pneumonia
10	Kim et al., 2007 [17]	63 y/F	Left lobe	Well-differentiated	Left lobe hepatectomy	n/a
11	Binesh et al., 2012 [18]	83 y/F	Left lobe	Myxoid	Left lobe hepatectomy and chemotherapy (1st time); left lobe hepatectomy (2nd time)	Local recurrence 19 months after 1st operation; NED after 2nd operation
12	Naik et al., 2013 [6]	42 y/M	Left lobe	Pleomorphic	Left lobe hepatectomy and chemoradiotherapy	NED
13	Liu et al., 2018 [19]	29 y/M	Left lobe, with intraperitoneal metastases	Myxoid	Chemotherapy	Death 5 months after initial diagnosis due to extensive metastasis
14	Present case	62 y/F	Right lobe	Pleomorphic	Partial right lobe hepatectomy (1st time); right hepatectomy (2nd time); chemotherapy	Local recurrence 41 months after 1st operation; distant metastases 1 month after 2nd operation; death 5 months after recurrence

*y* years, *F* female, *M* male, *n/a* not available, *NED* no evidence of disease

## Conclusions

We reported a case of pleomorphic liposarcoma originating from the liver. Liposarcoma arising from the liver is so rare that clinicians may struggle to reach a definitive diagnosis. Due to the high recurrence rate and highly malignant progression, further research regarding the etiology of this unusual and highly malignant clinical entity is warranted to establish effective diagnostic and management protocols.

## Data Availability

No datasets were generated or analyzed during the current study.

## Abbreviations

CT: Computed tomography
MRI: Magnetic resonance imaging
HCC: Hepatocellular carcinoma
T2WI: T2-weighted image
T1WI: T1-weighted image
FISH: Fluorescent in situ hybridization
WHO: World Health Organization
MAID: Mesna, adriamycin, ifosfamide, and dacarbazine
MSC: Mesenchymal stem cell
HPC: Hepatic progenitor cell
PEComa: Perivascular epithelioid cell tumor
GIST: Gastrointestinal stromal tumor
SFT: Solitary fibrous tumor
